# Metals in the Environment: Toxic Metals Removal

**DOI:** 10.1155/2017/4309198

**Published:** 2017-07-16

**Authors:** Muataz A. Atieh, Yun Ji, Viktor Kochkodan

**Affiliations:** ^1^Qatar Environment and Energy Research Institute (QEERI), Hamad Bin Khalifa University (HBKU), Qatar Foundation, Doha, Qatar; ^2^College of Science and Engineering, Hamad Bin Khalifa University, Qatar Foundation, P.O. Box 5825, Doha, Qatar; ^3^Chemical Engineering Department, College of Engineering & Mines (CEM), University of North Dakota, Grand Forks, ND, USA

Faced with more and more stringent environmental regulations, nowadays heavy metals are the priority pollutants of surface and ground waters. Water contamination with these compounds is becoming one of the most serious environmental problems because of the toxic nature of the heavy metal ions, even at low trace levels. With the rapid development of industries such as metal plating facilities, mining operations, tanneries, fertilisers, and paper industries, heavy metals wastewaters are directly or indirectly discharged into the environment increasingly. Unlike organic contaminants, heavy metals are not biodegradable and tend to accumulate in living organisms. Many heavy metal ions, such as mercury, cadmium, lead, nickel, and chromium, are known to be very toxic or carcinogenic. Due to the noxious effects of heavy metals, there are growing public health concerns about environmental pollution with heavy metals. Thus, it is imperative to remove or reduce heavy metal contamination in water in order to prevent or reduce contaminating the environment and the possibility of uptake in the food web. This issue contains original research studies on removal of heavy metals from water by different treatment methods including adsorption, membranes, and coagulation.

The paper by F. A. Olabemiwo et al. investigated the potential ability of raw fly ash (RFA) and polyelectrolyte-coated fly ash (PEFA) to remove cadmium (Cd) from polluted water and described the removal of cadmium (Cd) ions from polluted water using raw fly ash (RFA) and polyelectrolyte-coated fly ash (PEFA). They revealed that a 4.0 g/L dosage of PEFA removed around 99% of 2.0 mg/L of Cd in 15 min at 150 rpm compared to only 27% Cd removal achieved by RFA under the same conditions. A comparative study of raw and metal oxide impregnated carbon nanotubes for the adsorption of hexavalent chromium from aqueous solution was carried out by M. I. Qureshi et al. They reported the use of raw, iron oxide, and aluminum oxide impregnated carbon nanotubes (CNTs) for the adsorption of hexavalent chromium (Cr(VI)) ions from aqueous solution. They showed that impregnated CNTs achieved significant increase in the removal efficiency of Cr(VI) ions compared to raw CNTs. In fact, both CNTs impregnated with 10% loading of iron and aluminum oxides were able to remove up to 100% of Cr(VI) ions from aqueous solution. While O. Y. Bakather et al. studied the removal of selenium ions from aqueous solution using iron oxide impregnated carbon nanotubes (CNTs). Total removal of 1 ppm Se ions from water was achieved when 25 mg of CNTs impregnated with 20 wt.% of iron oxide nanoparticles is used. Maximum adsorption capacity of the Fe_2_O_3_ impregnated CNTs, predicted by Langmuir isotherm model, was found to be 111 mg/g. Also using Fe_2_O_3_ impregnated CNTs have shown an efficient removal of hazardous organic components such as toluene and paraxylene (p-xylene) from aqueous solution as reported by A. Abbas et al. Batch adsorption experiments show that iron oxide impregnated CNTs have higher degree of removal of p-xylene (i.e., 90%) compared with toluene (i.e., 70%), for soaking time 2 h, with pollutant initial concentration of 100 ppm, at pH 6 and shaking speed of 200 rpm at 25°C. This new finding might revolutionize the adsorption treatment process and application by introducing a new type of nanoadsorbent that has super adsorption capacity towards heavy metals ions and organic pollutants from water. In addition, S. N. A. Shah et al. cover the chemical as well as the biological concerns about nanoparticles (NPs) particularly titanium dioxide (TiO_2_) NPs and emphasize the toxicological profile of TiO_2_ at the molecular level in both in vitro and in vivo systems. Although nanoparticles (NPs) have made incredible progress in the field of nanotechnology and biomedical research and their applications are demanded throughout industrial world particularly over the past decades, little is known about the fate of nanoparticles in ecosystem. Concerning the biosafety of nanotechnology, nanotoxicity is going to be the second most priority of nanotechnology that needs to be properly addressed.

Another study was carried out by S. A. Zamani et al., to produce optimized biochar from oil palm empty fruit bunches (OPEFB), as a green, low cost adsorbent for uptake of zinc from aqueous solution. High removal of zinc was achieved by this green bioactive carbon material.

We hope that this special issue would shed light on many important aspects related to removal of heavy metals from water and attract attention by the scientific community to pursue further investigations in this field.

